# Mendelian Randomization Study Supports Genetic Liability to Obsessive–Compulsive Disorder Associated With the Risk of Alzheimer's Disease

**DOI:** 10.1002/brb3.70081

**Published:** 2024-09-30

**Authors:** Si Cao, Han Su, Xiaoyi Zhang, Chao Fang, Nayiyuan Wu, Youjie Zeng, Minghua Chen

**Affiliations:** ^1^ Department of Anesthesiology, Third Xiangya Hospital Central South University Changsha Hunan China; ^2^ Department of Medicine, Jacobi Medical Center Albert Einstein College of Medicine Bronx New York USA; ^3^ The Affiliated Cancer Hospital of Xiangya School of Medicine Central South University/Hunan Cancer Hospital Changsha Hunan China

**Keywords:** Alzheimer's disease, causal inference, obsessive–compulsive disorder, risk factors

## Abstract

**Background:**

Observational studies have suggested that obsessive–compulsive disorder (OCD) may be associated with Alzheimer's disease (AD). However, whether OCD is a causal risk factor for AD remains unclear. This study aimed to assess the causal effect of OCD on AD risk by performing a two‐sample Mendelian randomization (MR) analysis.

**Methods:**

Genome‐wide association summary statistics were obtained for OCD, comprising 2688 cases and 7037 controls, as well as for AD, including 21,982 cases and 41,944 controls from Kunkle et al.’s study, and 39,918 cases and 358,140 controls from Wightman et al.’s study. On the basis of two diverse thresholds, OCD‐associated genetic variants were screened as instrumental variables (IVs) for subsequent MR analyses. Inverse variance weighed was the primary MR method. MR‐Egger, weighted median, and weighted mode were used as supplementary MR methods. Various sensitivity tests assessed the reliability of MR results.

**Results:**

On the basis of strict IV selecting thresholds, inverse‐variance weighted (IVW) identified significant causal associations between genetic liability to OCD and increased risk of AD in two different sources ((i) Kunkle et al.: odds ratio [OR] = 1.070, 95% confidence interval [CI]: 1.015–1.127, *p = *0.012; (ii) Wightman et al. 0.012; (iii) Wightman et al.: OR = 1.051, 95% CI: 1.014–1.090, *p = *0.007). Three other supplementary MR methods yielded similar results to IVWs (OR > 1). Furthermore, all results were replicated in MR analyses based on lenient IV selecting thresholds. The sensitivity tests indicated that MR results were stable and not affected by significant horizontal pleiotropy.

**Conclusions:**

This comprehensive MR study suggests that genetic liability to OCD is a causal risk factor for AD. Early intervention in patients with OCD may be beneficial in preventing future AD progression.

## Graphical abstract

This study conducted a two‐sample Mendelian randomization (MR) analysis based on genome‐wide association study (GWAS) summary statistics for obsessive–compulsive disorder (OCD) and Alzheimer's disease (AD). Using various MR methods and sensitivity tests, the study ultimately indicates that genetic liability to OCD is causally associated with an increased risk of AD.

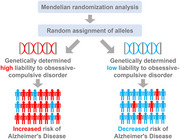

## 1 Introduction

Alzheimer's disease (AD) is a devastating, progressive neurodegenerative disorder marked by abnormal protein aggregation and neuronal loss, leading to cognitive decline, memory loss, and eventually death, making it the leading cause of dementia and one of the most fatal and challenging diseases worldwide (Scheltens et al. 2021). The pathological hallmarks of AD include amyloid‐beta plaques, neurofibrillary tangles, and widespread neuronal loss, which significantly impair daily functioning (DeTure and Dickson 2019). Over the past 30 years, with the demographic shift toward an aging population, the burden of AD has been continuously increasing, particularly among the elderly in areas/countries with high sociodemographic index (Li et al. 2022). Therefore, early intervention based on identifying and mitigating AD risk factors is crucial for preventing its progression (Crous‐Bou et al. 2017).

Obsessive–compulsive disorder (OCD) is a chronic psychiatric condition marked by intrusive thoughts (obsessions) and repetitive behaviors (compulsions) (Fenske and Petersen 2015). It typically manifests in adolescence or early adulthood and can cause substantial distress and impairment (Fenske and Petersen 2015). Recent studies have suggested a potential link between OCD and AD. An earlier study by Mrabet Khiari et al. (2011) reported a case where a 75‐year‐old diagnosed with AD had a history of OCD, which was also observed in several of their family members before they were later diagnosed with AD. Moreover, several studies indicated an association between OCD and AD (Chen et al. 2021; Dondu et al. 2015; Ruggeri et al. 2022). However, these findings were derived from observational research and may have been influenced by residual confounding and reverse causation. Therefore, it remains unclear whether OCD is a risk factor for AD.

Mendelian randomization (MR) study is an epidemiological method that leverages genetic variants as instrumental variables (IVs) to infer causal relationships between risk factors and disease outcomes (Sanderson et al. 2022). Single‐nucleotide polymorphisms (SNPs), the most commonly used IVs in MR studies, are utilized to represent different exposure statuses (cases and controls) (Bowden and Holmes 2019). Additionally, because SNPs are formed during meiosis based on distribution principles and are not easily influenced by postnatal environmental factors, MR studies can be likened to natural randomized controlled trials (RCTs) (Swanson et al. 2017). The extensive public availability of summary‐level statistics from genome‐wide association studies (GWASs) facilitates the execution of MR studies (Swerdlow et al. 2016). Given the undetermined causal relationship between OCD and AD, this study aimed to perform an MR analysis, assessing whether there was a causal link between genetic liability to OCD and an increased risk of AD.

## 2 Materials and Methods

### 2.1 Overall Study Process

**FIGURE 1:**
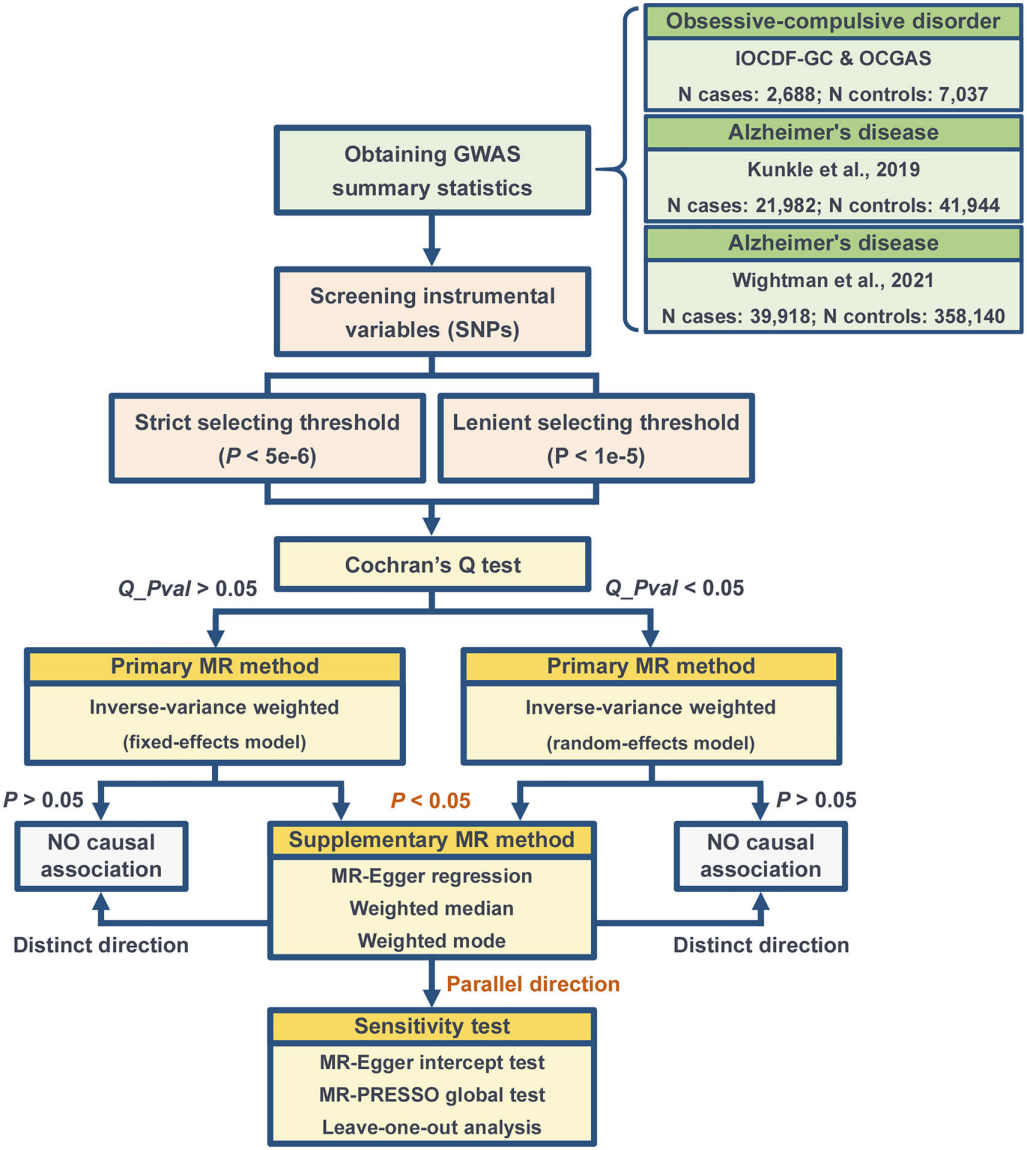
The overall flowchart of this MR study. GWAS, genome‐wide association study; IOCDF‐GC, International Obsessive Compulsive Disorder Foundation Genetics Collaborative; MR, Mendelian randomization; OCGAS, OCD Collaborative Genetics Association Studies.

All analyses were performed using the TwoSampleMR package in R software. Figure 1 illustrates the overall workflow of this MR study. This study employed a two‐sample MR analysis framework, using summary‐level GWAS statistics from OCD and AD for causal inference. First, summary‐level GWAS statistics for OCD and AD were downloaded from publicly accessible databases storing GWAS summary statistics. To enhance the reliability of the findings, summary statistics for AD from two different sources were obtained. Subsequently, on the basis of two different thresholds, IVs representing genetic susceptibility to OCD were selected for subsequent analysis. Various MR methods and sensitivity tests were then used to comprehensively assess the causal relationship between OCD and AD. Detailed information on each step is provided in the subsequent sub‐sections.

### 2.2 GWAS Summary Level Statistics for OCD and AD

The GWAS summary statistics for OCD are derived from a GWAS meta‐analysis conducted by the International Obsessive Compulsive Disorder Foundation Genetics Collaborative (IOCDF‐GC) and OCD Collaborative Genetics Association Studies (OCGAS) (2018). This study included a total of 2688 individuals of European descent with OCD and 7037 genomically matched controls. For the summary‐level statistics of AD, data from two different sources were included: (i) from a GWAS meta‐analysis by Kunkle et al. (2019), which included 21,982 AD cases and 41,944 controls; (ii) from a GWAS meta‐analysis by Wightman et al. (2021), providing summary statistics for 39,918 AD patients and 358,140 controls. Despite some sample overlap between the two AD datasets, using diverse AD summary statistics helps strengthen the credibility of the findings. Download addresses for all GWAS summary statistics included in this MR study are shown in Table S1.

### 2.3 Statistical Analysis

SNPs strongly associated with OCD were used as IVs proxying for OCD. Initially, SNPs with strong associations were filtered from the OCD GWAS summary statistics based on significance thresholds. This study employed two different thresholds: a strict threshold (*p* < 5e − 6) and a lenient threshold (*p* < 1e − 5) to select IVs. The strict threshold minimizes confounding factors, whereas the more lenient threshold allows for a greater number of IVs, enhancing the statistical efficiency. Furthermore, because previous studies have suggested that intelligence and education may influence both OCD and AD (Anderson et al. 2020; Baranova, Cao, and Zhang 2024), we aimed to ensure that our MR study was not affected by these confounding factors. We first obtained GWAS summary statistics for intelligence (Savage et al. 2018) and education (Okbay et al. 2022) (download link provided in Table S1) and identified all SNPs associated with these two phenotypes (*p* < 1e − 5) (Table S2). Any SNPs related to these two phenotypes would be excluded from IVs.

Inverse‐variance weighted (IVW) was the primary method for causal inference, integrating the final causal effect through a meta‐analysis of causal inferences made using the Wald ratio method based on individual IVs (Burgess, Butterworth, and Thompson 2013). Before conducting MR analysis, Cochran's *Q* test was performed to assess heterogeneity among causal inferences from different individual IVs. If no heterogeneity was present (*p* > 0.05), the fixed‐effects model–based IVW was used as the primary method. If heterogeneity was detected (*p* < 0.05), the random‐effects model–based IVW was adopted as the primary method. Additionally, three supplementary methods—MR‐Egger regression, weighted median, and weighted mode—were used to complement the IVW. Causal inference was presented through odds ratio (OR) and 95% confidence interval (CI). If the *p* value from the IVW estimation is less than 0.05 and the OR values from the three supplementary methods are consistent with the IVW, this indicates a significant causal association.

Finally, the reliability of the MR estimation was further assessed through various sensitivity tests. Horizontal pleiotropy refers to the possibility that IVs affect the outcome through paths other than the exposure. Horizontal pleiotropy was evaluated using the MR‐Egger intercept test and the MR‐PRESSO global test. A *p* value greater than 0.05 indicates that the MR analysis is not significantly influenced by horizontal pleiotropy. Additionally, the leave‐one‐out test, which involves repeatedly conducting MR analyses after sequentially removing each IV, can assess whether any outlier leading IVs significantly alter the MR results, thereby evaluating the stability of the estimation.

## 3 Results

### 3.1 Identification of IVs

Table S3 shows the details of the IVs used to assess the causal effect of OCD on AD under the strict IV selecting threshold. Specifically, a total of 10 and 11 IVs are used to assess the causal associations of OCD with AD from the studies by Kunkle et al. and AD from the studies by Wightman et al., respectively. Table S4 displays detailed information of the IVs used to assess the causal effect of OCD on AD based on the lenient IV selecting threshold. Specifically, 19 and 20 IVs are used for assessing the causal relationships between OCD and AD from the studies by Kunkle et al. and AD from the studies by Wightman et al., respectively. All identified IVs were not associated with the confounders of intelligence and educational attainment (Table S2). The *F*‐statistics of all IVs ranged from 19.57 to 25.57, indicating that the IVs were not subject to the bias of weak instruments.

### 3.2 Results of MR Analysis

Cochran's *Q* test showed that none of the IVs contribute to significant heterogeneity (Table S5), and therefore, the IVW with fixed‐effects model would be adopted as the primary method for causal inference.

**FIGURE 2:**
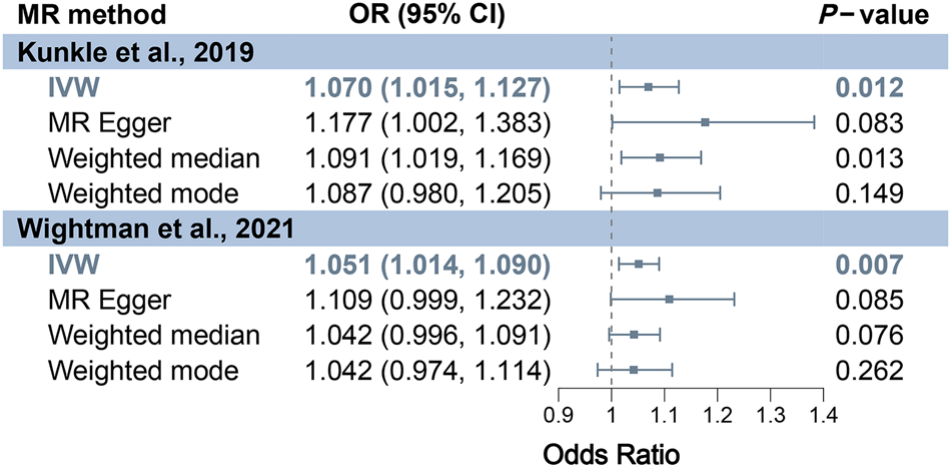
MR results for assessing the causal effect of OCD on AD based on the strict IV selecting threshold. CI, confidence interval; IVW, inverse‐variance weighted; MR, Mendelian randomization.

Figure 2 presents the MR results based on the strict IV selecting threshold for assessing the causal effect of OCD on AD. For AD from Kunkle et al., IVW showed that genetic liability to OCD significantly increases AD risk (OR = 1.070, 95% CI: 1.015–1.127, *p* = 0.012). Additional methods, including MR‐Egger (OR = 1.177), weighted median (OR = 1.091), and weighted mode (OR = 1.087), yielded parallel results (OR > 1). Moreover, for AD from Wightman et al., IVW indicated a significant increase in AD risk due to OCD (OR = 1.051, 95% CI: 1.014–1.090, *p* = 0.007), with MR‐Egger (OR = 1.109), weighted median (OR = 1.042), and weighted mode (OR = 1.042) showing parallel results (OR > 1).

**FIGURE 3:**
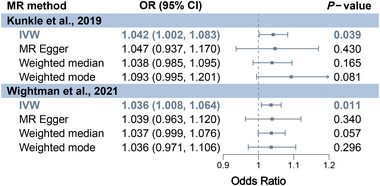
MR results for assessing the causal effect of OCD on AD based on the lenient IV selecting threshold. CI, confidence interval; IVW, inverse‐variance weighted; MR, Mendelian randomization.

Figure 3 shows the MR results based on the lenient IV selecting threshold. For AD from Kunkle et al., IVW showed that genetic liability to OCD significantly increases AD risk (OR = 1.042, 95% CI: 1.002–1.083, *p* = 0.039). Additional methods, including MR‐Egger (OR = 1.047), weighted median (OR = 1.038), and weighted mode (OR = 1.093), yielded parallel results (OR > 1). Moreover, for AD from Wightman et al., IVW demonstrated that OCD significantly heightens AD risk (OR = 1.036, 95% CI: 1.008–1.064, *p* = 0.011), with MR‐Egger (OR = 1.039), weighted median (OR = 1.037), and weighted mode (OR = 1.036) corroborating these findings (OR > 1).

**FIGURE 4:**
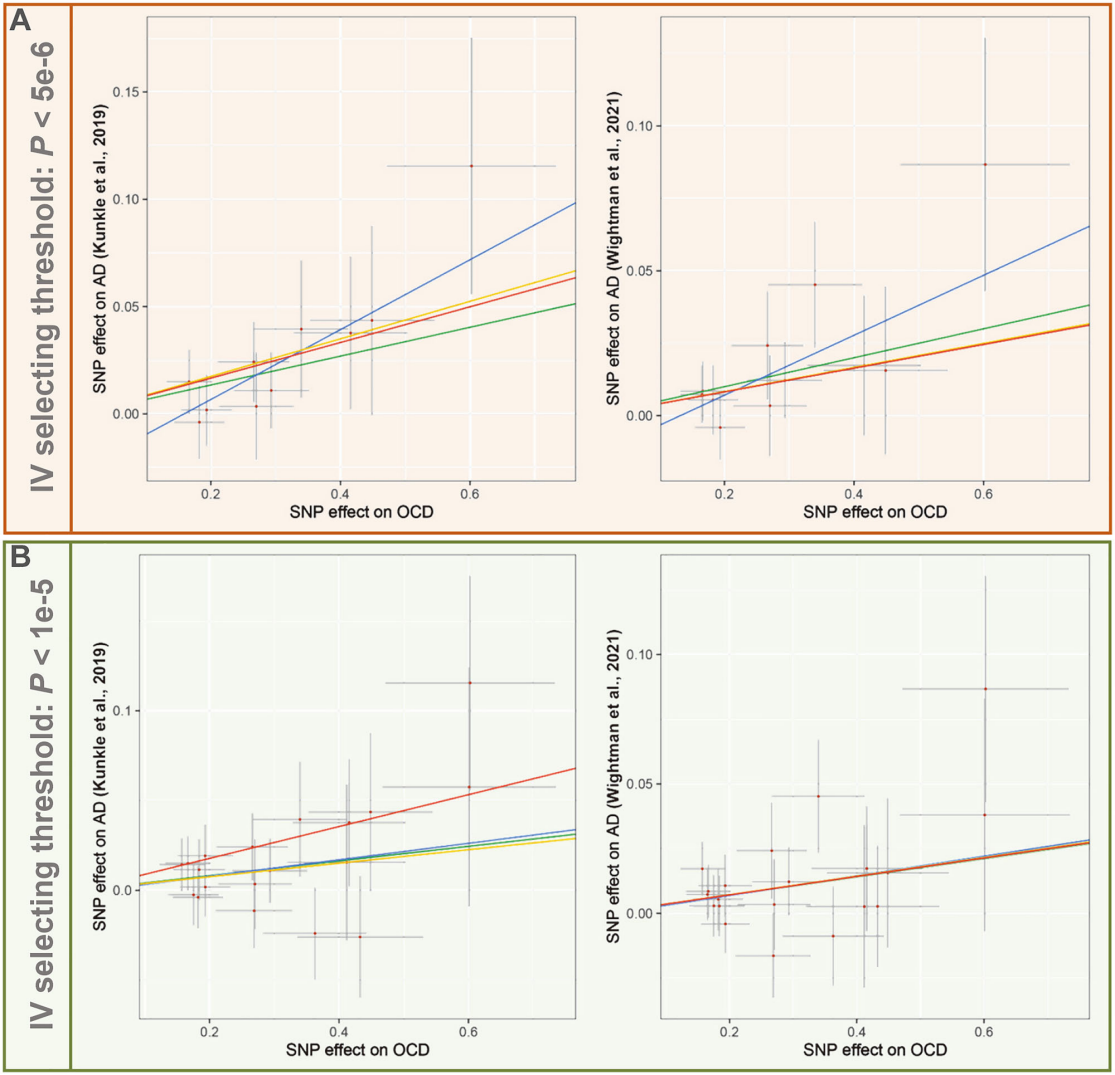
Scatter plot of IV association with each trait: (A) scatter plot of IV impacts on OCD versus AD based on the strict screening threshold; (B) scatter plot of IV impacts on OCD versus AD based on the lenient screening threshold. AD, Alzheimer's disease; IV, instrumental variable; OCD, obsessive–compulsive disorder; SNP, single‐nucleotide polymorphism.

Consequently, on the basis of two sets of IV selecting thresholds, genetic susceptibility to OCD was identified as a risk factor for AD across different sourced AD datasets. Figure 4 intuitively presents the IVs (SNPs) used for MR analysis and their associations with OCD and AD through a scatter plot, indicating a significant positive causal effect of OCD on the risk of AD.

### 3.3 Results of the Sensitivity Test

**FIGURE 5:**
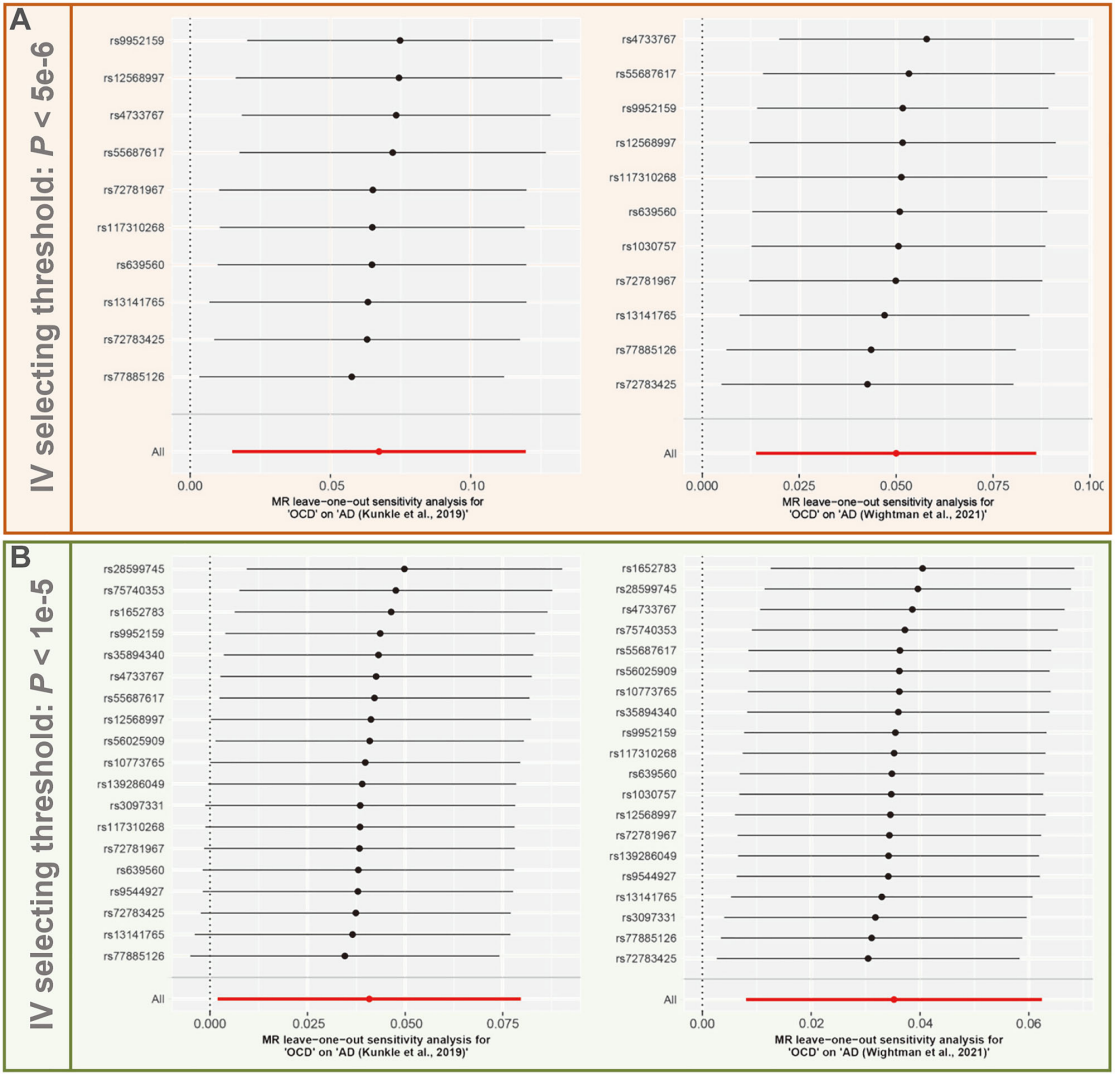
Results of leave‐one‐out analysis: (A) results of the leave‐one‐out test based on the strict IV screening threshold; (B) results of the leave‐one‐out test based on the lenient IV screening threshold. AD, Alzheimer's disease; IV, instrumental variable; OCD, obsessive–compulsive disorder; SNP, single‐nucleotide polymorphism.

Various sensitivity tests were performed to further assess the reliability of the MR findings. Table 1 demonstrates the results of the horizontal pleiotropy sensitivity test. Whether based on the strict IV selecting threshold or the lenient IV selecting threshold, both the MR‐Egger intercept test and the MR‐PRESSO global test did not detect significant horizontal pleiotropy (*p* > 0.05) in both sources of the AD dataset. In addition, the leave‐one‐out sensitivity test showed that no significant outlier SNPs were detected among the IVs screened based on both strict threshold (Figure 5A) and lenient threshold (Figure 5B), indicating the stability of the overall MR results.

**TABLE 1:** Results of the horizontal pleiotropy sensitivity test.

AD data source	MR‐Egger intercept test			MR‐PRESSO global test	
	Intercept	SE	*p* value	RSS obs	*p* value
**IV selecting threshold: *p *< 5e−6**					
Kunkle et al. (2019)	−2.59e − 02	0.021	0.254	4.914	0.923
Wightman et al. (2021)	−1.37e − 02	0.013	0.314	6.751	0.858
**IV selecting threshold: *p *< 1e−5**					
Kunkle et al. (2019)	−1.34e − 03	0.014	0.926	12.801	0.882
Wightman et al. (2021)	−6.93e − 04	0.009	0.941	13.464	0.886

Abbreviations: AD, Alzheimer's disease; IV, instrumental variable; MR, Mendelian randomization.

## 4 Discussion

To our knowledge, this is the first study to evaluate the causal association between the risk of OCD and AD based on MR analysis. On the basis of two different IV selecting thresholds, combined with two sets of AD datasets from different sources, through various MR methods and sensitivity tests, it comprehensively supported that genetic liability to OCD increased the incidence risk of AD.

Recent studies have emphasized the link between multiple psychiatric disorders and AD. First, a wide range of psychiatric symptoms are commonly observed in AD patients. For example, a meta‐analysis indicates that neuropsychiatric symptoms like apathy, depression, aggression, and anxiety are more prevalent in patients with AD (Zhao et al. 2016). Moreover, these psychiatric disorders are not only common in AD patients but also increase the risk of developing AD. Individuals with lifelong depression have a higher risk of developing AD (Caraci et al. 2010). Similarly, late‐life clinically significant anxiety has been shown to significantly increase the risk of AD, independent of depression and other confounders (Santabárbara et al. 2019).

Among the psychiatric conditions, the link between OCD and AD has garnered increasing research attention in recent studies. Despite the limited number of studies on this topic, some earlier observational studies have indicated the potential association between OCD and AD. A study based on the Taiwan National Health Insurance Research Database included 1347 OCD patients aged ≥45 years and 13,470 controls matched for age, gender, residence, income, and dementia‐related comorbidities, finding that the risk of developing AD was significantly higher in the OCD group than in the controls (hazard ratio = 4.04, 95% CI: 1.55–10.54) (Chen et al. 2021). Another study indicated that the mean number of lifetime (*p* < 0.0001) and current obsessions (*p* = 0.001) in the AD group was significantly higher than in the control group (Dondu et al. 2015). Additionally, the mean number of lifetime (*p* < 0.0001) and current compulsions (*p* = 0.002) were also greater in patients with AD compared to control subjects (Dondu et al. 2015). The present MR study further provided evidence of a causal association from the previous observational studies, emphasizing OCD as a causal risk factor for AD. Interestingly, a previous MR study also assessed the causal effect of OCD on AD (Baranova et al. 2024), and although their *p* values were not significant (*p* = 0.103)—likely due to the inclusion of proxy AD cases in the GWAS summary statistics—the similarity in OR (OR = 1.05) to our findings reinforces the consistency and potential validity of the association.

The results of this MR study need to be reasonably interpreted. First, all the OR values of the IVW results in this MR study ranged from 1.03 to 1.07, suggesting that compared to the control group, OCD patients have a 3%–7% higher risk of developing AD. Additionally, because SNPs occur during the formation of the zygote and are almost unaffected by the postnatal environment, the MR study reveals the effects of long‐term exposure. Therefore, in this MR study, it can be considered that the occurrence of AD is due to the long‐term accumulation of the OCD disease course, which is consistent with previous studies indicating a higher proportion of lifelong OCD in AD patients (Dondu et al. 2015).

The potential mechanisms through which genetic liability to OCD acts as a causal risk factor for AD have not been fully elucidated. However, several plausible pathways might explain this association. OCD and AD may share common upstream mechanisms, as recent studies have shown that dysregulation of insulin signaling is closely associated with the progression of both OCD and AD (Bralten et al. 2020; Butterfield and Halliwell 2019). In addition, recent proteomic analysis of serum from patients with OCD revealed interactions among proteins, such as apolipoprotein A‐4, haptoglobin, component 3, albumin, amyloid precursor protein, and protein α1‐antitrypsin, which are involved in inflammation, hydrogen peroxide catabolism, and triglyceride metabolism, suggesting potential molecular links between OCD and AD through shared inflammatory and oxidative stress pathways (Zamanian‐Azodi et al. 2017). Research shows that treatment‐refractory OCD patients, with decreased gray matter density in the left posterior cingulate cortex and mediodorsal thalamus and increased density in the left dorsal striatum, exhibit brain changes similar to those observed in AD, suggesting a common neuropathological pathway (Bernstein et al. 2021; Choo et al. 2010; Tang et al. 2013). Furthermore, studies have identified synaptic dysfunction in OCD patients (Xiao et al. 2024), and this dysfunction is known to contribute to the development of AD (Meftah and Gan 2023). In addition, a recent research has found that the APOE ɛ4ɛ4 genotype is more prevalent in OCD patients, which may also contribute to the higher risk of AD in these individuals (Dondu, Caliskan, and Orenay‐Boyacioglu 2024). Nevertheless, the specific mechanism linking OCD and AD remains to be determined in future investigations.

This study has several advantages. First, it was the first time that large‐scale GWAS summary statistics were utilized to assess the causal association between the risk of OCD and AD through two‐sample MR analysis. This overcame the limitations of small sample sizes in previous observational studies and enhanced efficiency. Second, this MR study, within the framework of traditional two‐sample MR research, used diverse IV selecting thresholds and combined summary statistics from two different sources of outcomes to obtain consistent results, thereby enhancing the reliability of the findings.

It must be acknowledged that this study has some limitations. First, despite using two different IV selection thresholds, the limited number of available IVs prevented selection based on the genome‐wide significance threshold (*p* < 5e − 8). Nevertheless, various horizontal pleiotropy tests were conducted to ensure the reliability of the IVs. Additionally, due to the unavailability of individual‐level statistics and reliance only on summary‐level statistics, further subgroup analysis, such as evaluating the impact of OCD in early life on the future risk of AD, could not be performed. Therefore, further analysis based on more extensive GWAS summary statistics is necessary in the future.

## 5 Conclusion

This comprehensive MR study suggests that genetic liability to OCD is a causal risk factor for AD. Early intervention in patients with OCD may be beneficial in preventing future AD progression.

## Author Contributions

**Si Cao**: conceptualization, methodology, formal analysis, investigation, data curation, writing—original draft, visualization. **Han Su**: conceptualization, methodology, validation, investigation, writing—original draft, visualization. **Xiaoyi Zhang**: data curation, visualization, software, writing—review and editing. **Chao Fang**: software, data curation, writing—review and editing. **Nayiyuan Wu**: validation, data curation, writing—review and editing. **Youjie Zeng**: conceptualization, methodology, validation, formal analysis, investigation, writing—review and editing, project administration. **Minghua Chen**: conceptualization, methodology, validation, investigation, resources, writing—review and editing, supervision, project administration, funding acquisition.

## Ethics Statement

This study is a secondary analysis based on publicly available data from public databases, and thus does not require ethical approval.

## Consent

The authors have nothing to report.

## Conflicts of Interest

The authors declare no conflicts of interest.

### Peer Review

The peer review history for this article is available at https://publons.com/publon/10.1002/brb3.70081.

## Supporting information



Table S1 Details of GWAS summary‐level statistics for the present MR analysis.
Table S2 Details of SNPs associated with confounders (intelligence and educational attainment) (*p* < 1e − 5).
Table S3 Details of the IVs for assessing the causal effect of OCD on AD based on a strict IV selecting threshold (*p* < 5e − 6).
Table S4 Details of the IVs for assessing the causal effect of OCD on AD based on a lenient IV selecting threshold (*p* < 1e − 5).
Table S5 Results of Cochran's *Q* test.

## Data Availability

The datasets used in the present MR study are available in Table S1.
